# A high-resolution haplotype-resolved Reference panel constructed from the China Kadoorie Biobank Study

**DOI:** 10.1093/nar/gkad779

**Published:** 2023-10-23

**Authors:** Canqing Yu, Xianmei Lan, Ye Tao, Yu Guo, Dianjianyi Sun, Puyi Qian, Yuwen Zhou, Robin G Walters, Linxuan Li, Yunqing Zhu, Jingyu Zeng, Iona Y Millwood, Ruidong Guo, Pei Pei, Tao Yang, Huaidong Du, Fan Yang, Ling Yang, Fangyi Ren, Yiping Chen, Fengzhen Chen, Xiaosen Jiang, Zhiqiang Ye, Lanlan Dai, Xiaofeng Wei, Xun Xu, Huanming Yang, Jian Wang, Zhengming Chen, Huanhuan Zhu, Jun Lv, Xin Jin, Liming Li

**Affiliations:** Department of Epidemiology and Biostatistics, School of Public Health, Peking University Health Science Center, Beijing 100191, China; Center for Public Health and Epidemic Preparedness and Response, Peking University, Beijing 100191, China; Key Laboratory of Epidemiology of Major Diseases (Peking University), Ministry of Education, Beijing 100191, China; College of Life Sciences, University of Chinese Academy of Sciences, Beijing 100049, China; BGI Research, Shenzhen 518083, China; BGI Research, Shenzhen 518083, China; National Center for Cardiovascular Diseases, Fuwai Hospital, Chinese Academy of Medical Sciences, Beijing 100037, China; Department of Epidemiology and Biostatistics, School of Public Health, Peking University Health Science Center, Beijing 100191, China; Center for Public Health and Epidemic Preparedness and Response, Peking University, Beijing 100191, China; Key Laboratory of Epidemiology of Major Diseases (Peking University), Ministry of Education, Beijing 100191, China; China National GeneBank, BGI, Shenzhen 518083, China; College of Life Sciences, University of Chinese Academy of Sciences, Beijing 100049, China; BGI Research, Shenzhen 518083, China; Clinical Trial Service Unit and Epidemiological Studies Unit (CTSU), Nuffield Department of Population Health, University of Oxford, Oxford OX3 7LF, United Kingdom; Medical Research Council Population Health Research Unit, Nuffield Department of Population Health, University of Oxford, Oxford OX3 7LF, United Kingdom; College of Life Sciences, University of Chinese Academy of Sciences, Beijing 100049, China; BGI Research, Shenzhen 518083, China; Department of Epidemiology and Biostatistics, School of Public Health, Peking University Health Science Center, Beijing 100191, China; BGI Research, Shenzhen 518083, China; College of Life Sciences, Northwest A&F University, Yangling, Shaanxi 712100, China; Clinical Trial Service Unit and Epidemiological Studies Unit (CTSU), Nuffield Department of Population Health, University of Oxford, Oxford OX3 7LF, United Kingdom; Medical Research Council Population Health Research Unit, Nuffield Department of Population Health, University of Oxford, Oxford OX3 7LF, United Kingdom; BGI Research, Shenzhen 518083, China; Center for Public Health and Epidemic Preparedness and Response, Peking University, Beijing 100191, China; China National GeneBank, BGI, Shenzhen 518083, China; Clinical Trial Service Unit and Epidemiological Studies Unit (CTSU), Nuffield Department of Population Health, University of Oxford, Oxford OX3 7LF, United Kingdom; Medical Research Council Population Health Research Unit, Nuffield Department of Population Health, University of Oxford, Oxford OX3 7LF, United Kingdom; China National GeneBank, BGI, Shenzhen 518083, China; Clinical Trial Service Unit and Epidemiological Studies Unit (CTSU), Nuffield Department of Population Health, University of Oxford, Oxford OX3 7LF, United Kingdom; Medical Research Council Population Health Research Unit, Nuffield Department of Population Health, University of Oxford, Oxford OX3 7LF, United Kingdom; China National GeneBank, BGI, Shenzhen 518083, China; Clinical Trial Service Unit and Epidemiological Studies Unit (CTSU), Nuffield Department of Population Health, University of Oxford, Oxford OX3 7LF, United Kingdom; Medical Research Council Population Health Research Unit, Nuffield Department of Population Health, University of Oxford, Oxford OX3 7LF, United Kingdom; China National GeneBank, BGI, Shenzhen 518083, China; College of Life Sciences, University of Chinese Academy of Sciences, Beijing 100049, China; BGI Research, Shenzhen 518083, China; China National GeneBank, BGI, Shenzhen 518083, China; China National GeneBank, BGI, Shenzhen 518083, China; China National GeneBank, BGI, Shenzhen 518083, China; BGI Research, Shenzhen 518083, China; Guangdong Provincial Key Laboratory of Genome Read and Write, BGI Research, Shenzhen 518083, China; BGI Research, Shenzhen 518083, China; Guangdong Provincial Academician Workstation of BGI Synthetic Genomics, BGI, Shenzhen 518083, China; James D. Watson Institute of Genome Sciences, Hangzhou 310013, China; BGI, Shenzhen 518083, China; Clinical Trial Service Unit and Epidemiological Studies Unit (CTSU), Nuffield Department of Population Health, University of Oxford, Oxford OX3 7LF, United Kingdom; Medical Research Council Population Health Research Unit, Nuffield Department of Population Health, University of Oxford, Oxford OX3 7LF, United Kingdom; BGI Research, Shenzhen 518083, China; Center for Public Health and Epidemic Preparedness and Response, Peking University, Beijing 100191, China; State Key Laboratory of Vascular Homeostasis and Remodeling, Peking University, Beijing 100191, China; BGI Research, Shenzhen 518083, China; School of Medicine, South China University of Technology, Guangzhou 510006, China; Department of Epidemiology and Biostatistics, School of Public Health, Peking University Health Science Center, Beijing 100191, China; Center for Public Health and Epidemic Preparedness and Response, Peking University, Beijing 100191, China; Key Laboratory of Epidemiology of Major Diseases (Peking University), Ministry of Education, Beijing 100191, China

## Abstract

Precision medicine depends on high-accuracy individual-level genotype data. However, the whole-genome sequencing (WGS) is still not suitable for gigantic studies due to budget constraints. It is particularly important to construct highly accurate haplotype reference panel for genotype imputation. In this study, we used 10 000 samples with medium-depth WGS to construct a reference panel that we named the CKB reference panel. By imputing microarray datasets, it showed that the CKB panel outperformed compared panels in terms of both the number of well-imputed variants and imputation accuracy. In addition, we have completed the imputation of 100 706 microarrays with the CKB panel, and the after-imputed data is the hitherto largest whole genome data of the Chinese population. Furthermore, in the GWAS analysis of real phenotype height, the number of tested SNPs tripled and the number of significant SNPs doubled after imputation. Finally, we developed an online server for offering free genotype imputation service based on the CKB reference panel (https://db.cngb.org/imputation/). We believe that the CKB panel is of great value for imputing microarray or low-coverage genotype data of Chinese population, and potentially mixed populations. The imputation-completed 100 706 microarray data are enormous and precious resources of population genetic studies for complex traits and diseases.

## Graphical Abstract

**Graphical Abstract:**
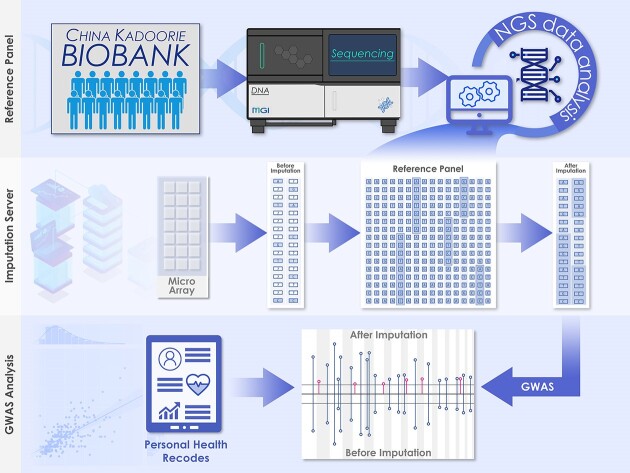

## Introduction

In recent years, precision medicine has made remarkable achievements in complex diseases retreatment and development of target drugs by using molecular biological information (e.g. individual genome) and clinical symptoms (1,2). Precision medicine relies on high-throughput whole-genome data to implement individual-based clinical diagnosis and treatment for patients. However, although the cost of whole-genome sequencing (WGS) technology has been greatly reduced, there is still a budget problem for large-scale population research. Most researchers still prefer the low-cost microarray-based genotyping technology, which sequences known loci to obtain genotype data for follow-up analysis. But microarray cannot mine novel mutation sites related to the disease, so there are limitations in the interpretation of the genetic mechanism of the disease. At present, the common method is to impute the microarray data at the whole-genome level based on the appropriate reference panel thus to obtain the whole-genome data for a population. The selection of reference genome plays an important role in the imputation accuracy of genome data and subsequent analysis results.

Internationally, the haplotype map (HapMap) (3,4), 1000 Genomes Project (1KGP) (5,6), the Haplotype reference consortium (HRC) (7) and trans-omics for precision medicine (TOPMed) (8) have been launched. The HapMap project is the next major human genomic program after the International Human Genome Project. In 2007, the HapMap (phase 3) sequenced 1184 individuals from 11 populations. In 2015, American, British and Chinses scientists jointly announced the completion of the Thousand Human Genome Project (phase 3), which sequenced the whole genomes of 2504 individuals from 26 global populations and created the most comprehensive genetic polymorphism map of the human genome. The 1KGP panel is the most-commonly used genome data to date. Recently, the expanded 1KGP cohort including 602 trios were published, in which all 3202 samples were sequenced to a high depth of 30 times (6). In 2016, the HRC project integrated 20 studies, such as UK10K and 1KGP, and created a reference panel with 32 470 individuals mostly with low-coverage WGS data (7). The latest TOPMed reference panel collected 97 256 individuals, including 47 159 Europeans, 24 267 Africans, 17 085 admixed Americans, 1184 East Asians, 644 South Asians and other populations (8).

In recent years, in addition to the international haplotype reference projects, national haploid genome sequence consortiums have also been initiated in various countries, including Netherlands, Denmark, Iceland and Singapore. The Dutch Human Genome Project sequenced 250 pedigrees at moderate depth (12×) to construct haploid reference sequences, substantially improving the accuracy of genotype inference for low-frequency variants (9). The Danish Genome Project sequenced 50 Danish families at high-depth (80×) WGS to construct the first Danish genome-wide high-precision haplotype reference panel (10). The Icelandic Genome performed high-depth (20×) WGS on ∼2000 individuals to create haplotype reference sequences, significantly improving the efficacy of association analysis and complex disease studies (11). The SG10K reference panel sequenced 4810 individuals, including 2780 Chinese, 903 Malays and 1127 Indians, with an average sequencing depth of 13.7× (12). This database is a valuable resource to advance the genetic study of complex traits and diseases in Asians.

China has the largest population in the world, producing enormous genetic resources, and should make a greater contribution to human genetics and complex disease research. However, the lack of high-quality haplotype reference sequences has become a bottleneck in the fields of population genetics and molecular biology. Fortunately, in the past 2 years, researchers have constructed reference panels based on Chinese population: the ChinaMAP (China Metabolic Analytics Project) and the Nyuwa reference panels. The ChinaMAP consortium performed 40× deep WGS on 10588 individuals collected from different regions and ethnicities in China (13,14). The library construction and WGS were performed on the BGISEQ-500 platform at BGI-Genomics. The ChinaMAP reference panel is a high-quality genetic variation database of Chinese population and plays an essential role in the analysis of Chinese population structure, genetic variation spectrum and pathogenic variants. The NyuWa reference panel includes 2902 independent samples with high-depth (26.2×) WGS collected from 23 administrative regions of China (15). It is important to expand the diversity of genetic resources and improve the accuracy of medical research in Chinese population.

The China Kadoorie Biobank (CKB), previously known as the Kadoorie Study of Chronic Disease in China (KSCDC), is an international collaborative research project on chronic diseases jointly conducted by Peking University, Chinese Academy of Medical Sciences and University of Oxford, UK (16). It is a gargantuan prospective study and the largest Chinese population cohort to date. During 2004–2008, >510 000 adults were recruited from 10 geographically defined regions in China. The study aims to establish a database of blood samples and clinical information and to investigate the main genetic and environmental causes of common chronic diseases. To date, the CKB cohort has achieved numerous influential findings in clinical studies, such as the relationship between smoking, physical activity, fresh fruit intake, egg consumption and the risk of cardiovascular disease (17–20), the association between diabetes and the risk of death (21) and the relationship between smoking, alcohol and tea consumption and esophageal cancer (22). However, unfortunately, there are no large-scale population genetics and genetic background studies of complex traits and diseases based on the CKB cohort (23). A major reason is the lack of high-density genetic data. Although microarray testing (Affymetrix Axiom myDesign) of >100 000 samples has been completed, the data are still not comparable to WGS data in terms of the number of genetic variants and the detection of novel loci.

In this work, we constructed a high-resolution haplotype-resolved reference panel based on 9950 individuals from the CKB cohort and 50 Chinese samples from the 1KGP study, with an average sequencing depth of 15.41×. We evaluated the imputation performance of the CKB reference panel from the perspective of number of imputed variants and imputation accuracy. The compared reference panels include the extended high coverage 1KGP, the newly developed TOPMed, the ChinaMAP and the NyuWa panels built from the Chinese population. In addition, based on the constructed CKB panel, we completed the genotype imputation for 100 706 microarray samples and obtained the largest whole genome data in the Chinese population. We further performed the genome-wide association study (GWAS) of human height based on the 100 706 microarray data before and after imputation. The total number of SNPs used in GWAS tripled after imputation and the number of significant loci increased from 119 to 147, while 26 out of the additional 28 identified loci were previously reported to be associated with height. We also created an online imputation server to offer free genotype imputation service (https://db.cngb.org/imputation/).

## Materials and methods

### Subjects

In this project, we constructed a haplotype reference panel based on 10 000 Chinese individuals, including 9950 from the CKB cohort and 50 from the 1KGP Han Chinese. The CKB project recruited >510 000 adults aged from 30 to 79 in 10 (five urban, five rural) geographic regions of China. These 9950 individuals were stroke cases from the cohort. The 50 1KGP samples included 20 northern and 30 southern Han Chinese. We also used 100 706 CKB microarray samples (independent of the 9950 samples) in subsequent analyses. Written informed consent was obtained from all participants from the CKB cohort.

### DNA samples and library construction

The WGS was performed for the 10 000 samples. Specifically, DNA concentration was measured by ExKubit dsDNA HS Assay Kits (Shanghai ExCell Biology, Inc) and Fluostar Omega Microplate Reader (BMG Labtech GmbH). The DNA quality was evaluated by agarose gel electrophoresis at a constant voltage (180 V) for 35 min. The DNA shearing was done by the Covaris E220 ultrasonics DNA shearing instruments. The DNA purification and fragment size selection were applied by VAHTS DNA Clean Beads (Vazyme, #N411). The libraries were constructed on BGI’s DNBseq-T1 × 4RS platform and the loading DNA concentration was >12 ng/µl. The paired-end 100-bp (PE100) WGS with 350-bp insert sizes was performed on the MGI DNBSEQ sequencing platform.

### Variant calling and sample quality control

To perform variant calling on each sample (also known as individual variant calling), we first applied SOAPnuke (v.2.1.1; -n 0.1 -l 12 -M 2) (24) to filter low quality reads and remove adapter sequences. Then, we obtained aligned Binary Alignment/Map (BAM) files by aligning sequence reads to the GRCh38 human reference genome assembly with Sentieon (v.202010.04) bwa-mem algorithm (https://www.biorxiv.org/content/10.1101/115717v2). On the sorted and aligned BAM files, we used Sentieon drivers LocusCollector to collect information on duplicates and Dedup to remove the duplicates. For regions that contain insertions or deletions (INDELs), we further performed local realignment around INDELs to correct for mapping errors and increase the quality of INDEL detection by using the Sentieon Realigner algorithm. To increase the accuracy of variant calling, we carried out base quality score recalibration (BQSR) to BAM files based on the Sentieon QualCal algorithm, which created a recalibration table. This table file was then applied as an input to Sentieon Haplotyper for single-nucleotide polymorphisms (SNPs) and INDELs detection. After all these steps, we obtained the called variant sites for each sample in gVCF format. Note that, for this variant calling workflow, we used the Sentieon DNASeq toolkit instead of the GATK best practice (25) for the following reasons: (1) the DNASeq and GATK have near-identical variant detection accuracy, (2) the DNASeq is >30 times faster than GATK and (3) the DNASeq may be more suitable for less deeply sequenced samples (https://www.biorxiv.org/content/10.1101/115717v2).

Before performing joint variant calling, we first selected samples with (1) no evidence of contamination (VerifyBamID FREEMIX <0.03) (26), (2) high library quality measured by reads duplication rate <0.05, (3) mean sequencing depth ≥10× and (4) GC content between 40 and 44. The joint variant calling was then performed by GVCFtyper algorithm implemented in Sentieon, followed by variant quality score recalibration (VQSR) for SNPs and INDEls separately using GATK (27). In this way, we first built the models with VariantRecalibrator and then applied it in ApplyVQSR. After that, ExcessHet >54.69 and low-quality sites that did not pass VQSR were filtered out by SelectVariants. Finally, we calculated genotype posterior probabilities by CalculateGenotypePosteriors.

### Reference panel construction

**Figure 1.**
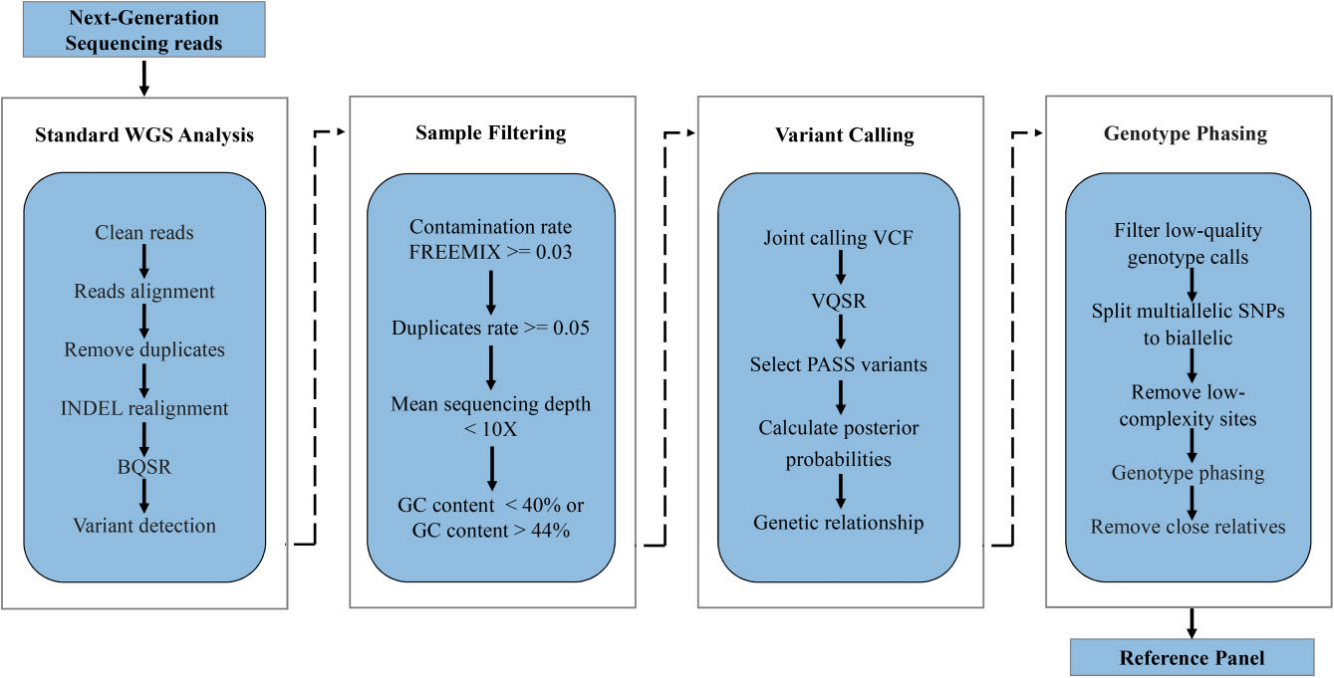
The workflow of panel construction.

After calculated genotype posterior probabilities, we further set low quality genotypes (GQ < 20) as missing and then removed low-complexity sites with minimum count of less than one or with missing alternate (ALT) allele. We also split a multiallelic SNP with more than one ALT allele to biallelic SNPs, with each ALT allele in a separate row. Next, we performed genotype phasing (also known as phasing/haplotype estimation), which is the process of statistical estimation of haplotypes from genotype data. This step was done by Beagle v.5.2 (28). Note that, during these steps, we did not remove related samples since the genetic relatedness can be modeled and improve haplotype phase accuracy. This concept was borrowed from the generation of the latest version of the 1000 Genome Project reference panel, in which the phasing accuracy was evaluated between inclusion and exclusion of trios; and the evaluation result showed that phasing with pedigree data achieved higher accuracy compared to unrelated samples alone (6). Finally, we removed close relatives up to the second degree generated by KING v.2.2.7 (29) as the related samples can distort the population allele frequency estimation in the subsequent analysis. After then, we obtained the reference panel, which we named as CKB reference panel. The construction workflow is provided in Figure 1.

We performed annotation analysis with the Ensembl Variant Effect Predictor (VEP) (30) by using plug-ins SIFT (31) and PolyPhen (32) algorithms. Additionally, we used ClinVar (33), (34) to label pathogenic variants and their related diseases in the ClinVar database. We kept variants only when its reference allele and alternate allele were consistent with that of CLINHGVS, which is a new INFO tag that reports the top-level genomic HGVS (Human Genome Variation Society) expression for the variant. We further calculated the alternate allele frequency (AF) as AF = AC/AN, where AC is the alternate allele count and AN is the total number of alleles.

### Evaluation of the imputation performance

We conducted extensive scenarios to evaluate the imputation performance of the CKB panel and others, including the extended 1KGP, TOPMed, ChinaMAP and NyuWa reference panels. There were two datasets to be imputed: the CKB microarray data and the 1KGP microarray data. From the CKB cohort, 50 randomly selected samples independent of that in the CKB reference panel were genotyped in both SNP array and high coverage WGS (44.14×). The 50 1KGP microarray samples were all Chinese and also independent of those in the CKB reference panel. To evaluate the imputation performance, we compared number of imputed variants and imputation accuracy. For the imputed variants, we defined high-quality variants with an imputed information score >0.8 and medium-quality variants with an imputed information score between 0.4 and 0.8. For the imputation accuracy, we calculated Pearson correlation coefficient (*R*^2^), precision and sensitivity. The high coverage WGS data were treated as ground truth when computing imputation accuracy between the imputed and true genotypes. For the CKB and extended 1KGP panels, we performed imputation procedures locally; while for TOPMed, ChinaMAP and NyuWa, we submitted jobs to their online imputation servers and downloaded the after-imputed files.

To assess the precision and sensitivity, we first calculated the true positive (TP), false positive (FP), false negative (FN) and true negative (TN). The TP indicates that the imputed genotype correctly predicts the true WGS genotype. The FP is an error classification where the imputed genotype incorrectly indicates the presence of a WGS variant. The FN is also an error classification where the imputed genotype incorrectly indicates the absence of a WGS variant. The TN is an outcome where the predicted genotype correctly predicts the case of homozygous reference calls. The details of 3 × 3 confusion matrix of defining TP, FP, FN and TN were provided in Supplementary Table S1. To eliminate the bias caused by the number of imputed variants, we compared the ratios of TP, FP, and FN instead of their counts directly. The TN value for all panels was zero. The ratio of TP was calculated by TP/(TP + FP + FN + TN), same for FP and FN. The precision was computed by TP/(TP + FP) and the sensitivity was computed by TP/(TP + FN).

### Imputation for 100 706 microarray data

We imputed 100 706 CKB microarray data based on the CKB reference panel in Beagle v.5.2 (28). Note that, the 50 samples with both microarray and high-coverage WGS data were included in the 100 706 individuals. To carry out imputation efficiently, we randomly divided the 100 706 samples into 21 chunks, in which 20 chunks contained 4800 samples and one chunk contained 4706 samples. Then, we parallelly executed genotype imputation for these chunks. To assess the performance for imputing such a large volume of data using the developed CKB panel, we extracted the 50 after-imputed microarray samples and calculated the Pearson correlation coefficients with their high coverage WGS set. We also compared this imputation accuracy with that of imputing the 50 microarray samples alone.

### PCA of the CKB reference panel and 100706 microarray data

To detect population stratification, we carried out principal component analysis (PCA) (35,36) of genotype data in the CKB reference panel. The PCA was carried out in Plink v.1.9 (37) with autosomal biallelic SNPs satisfying the following conditions (1) MAF ≥1%, (2) genotyping rate ≥90%, (3) Hardy--Weinberg equilibrium (HWE) *P*-value >1E-06 and (4) low linkage disequilibrium (LD, *r*^2^ < 0.5) with other variants in windows of 50 SNPs with steps of five SNPs. In addition, we performed PCA for 100 706 microarray data before imputation. The Plink arguments were the same as used previously.

### GWAS analysis of simulated data

In this section, we aimed to perform GWAS of simulated phenotypic values, whereas the genotype data were a combination of the CKB reference panel and after-imputed 100 706 microarray data. First, we performed PCA of genotype data by using PCAone (https://github.com/Zilong-Li/PCAone), which was applicable for large samples. Then, we simulated phenotype data under null and alternative hypotheses, separately. Under the null hypothesis that none of the SNPs were associated with the phenotype, we generated a vector of phenotypic values from a standard normal distribution. Under the alternative hypothesis that the phenotype data was generated from a linear regression model by using five SNPs as independent variables with randomly assigned effects size *β*. The causal SNPs included rs3003378 (*β* = 0.02), rs6764623 (*β* = 0.01), rs10905649 (*β* = 0.02), rs13254191 (*β* = 0.03) and rs10915307 (*β* = 0.01). We used PC1 to PC5 and sex of the participants as the covariates to carry out GWAS analysis in Plink v.2.0 (38). We reported Manhattan plots, QQ plots, histograms and regional plot for the GWAS results.

### GWAS analysis of real phenotype data

In this section, we performed GWAS analysis of real phenotype height, while the genotype data was 100 706 microarray data before and after imputation, separately. The covariates included age, sex, sampling site and the first 10 principal components of the microarray data before imputation. We used Plink v.2.0 for GWAS analysis by testing SNPs with MAF >0.01, HWE *P*-value >1E-06 and genotype missing rate <0.01. For the GWAS results, we defined a SNP as significant if its *P*-value >5E-08. We further grouped these significant SNPs into different loci by sliding a fixed-width (1 MB) window. For two loci identified before and after imputation, if the distance between their centers is within 500 KB, we defined that they were a shared locus.

### Online imputation service

We developed an online imputation server to offer genotype imputation service, which allows users to run imputation tasks free and safely in an easy way. For the online server, we provided the CKB and 1KGP as available reference panels, GRCh37 (hg19) and GRCh38 (hg38) as human genome assembly, Minimac v.4 (39,40) and Beagle v.5.2 (41) as imputation tools, and different population options. Specifically, for the CKB panel, Chinese is the sole population, and for the 1KGP panel, the available populations include East Asian, South Asian, African, European, Admixed American and all populations. Users can access the server via https://db.cngb.org/imputation/.

## Results

### Data quality

**Figure 2.**
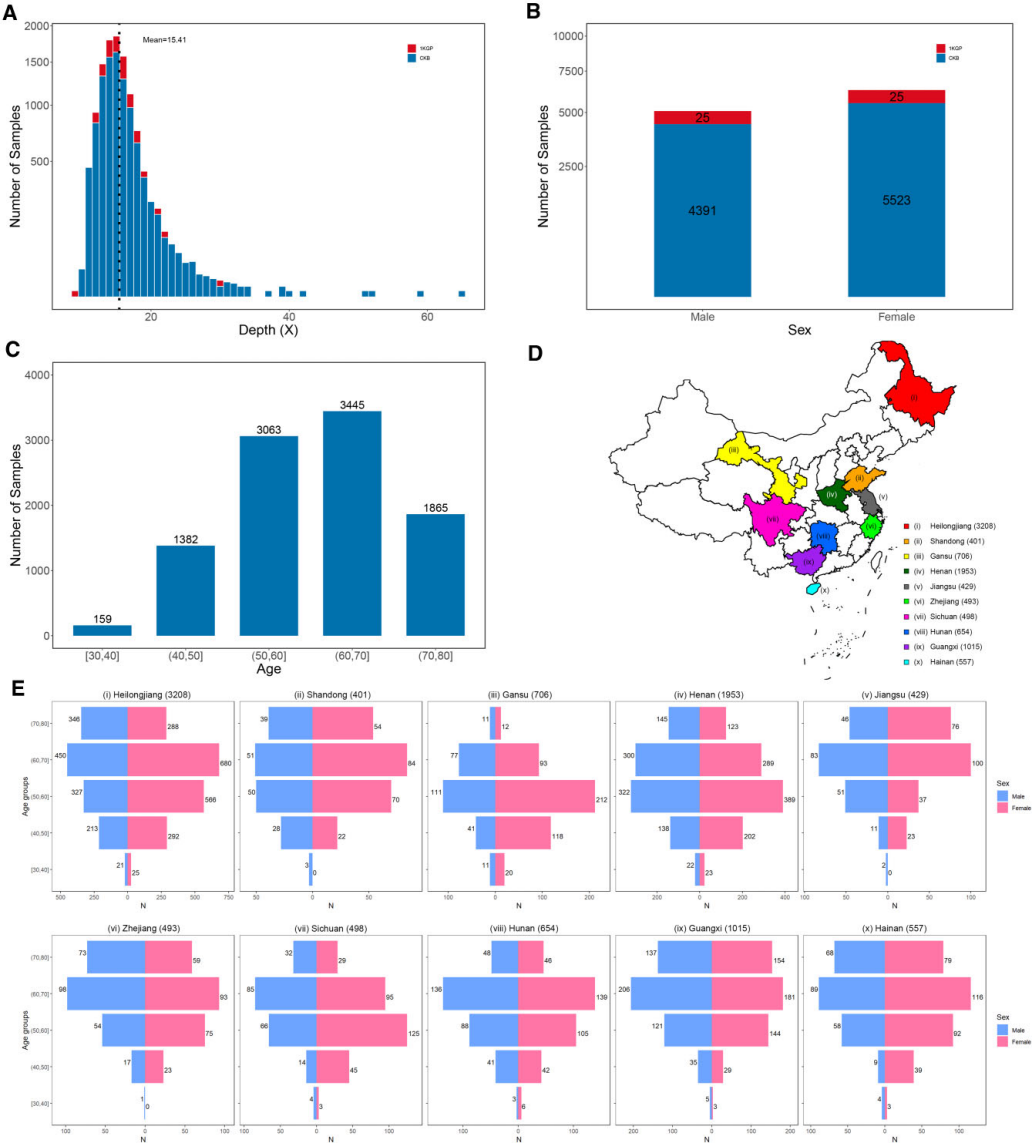
The sample information in the CKB reference panel. (**A**) The sequencing depth distribution of 50 1KGP samples (red) and 9914 CKB samples (blue). The mean sequencing depth of all 9964 samples was 15.41×. (**B**) The sex distribution of 50 1KGP samples (red) and 9914 CKB samples (blue). (**C**) The age distribution of 9914 CKB samples. (**D**) The China map colored for 10 sampling sites with number of samples. The total number of samples from all sampling sites was 9914. (**E**) The sex and age distributions of samples in each sampling site.

After sample-level quality control, the haplotype reference panel included 9964 individuals, where 9914 were from the CKB cohort and 50 were 1KGP Chinese. The sequencing depth, sex distribution, and age distribution are provided in Figure 2a--c. In detail, the mean sequencing depth was 15.41 (15.41 for CKB samples and 15.78 for 1KGP samples). There were 4416 males (44.32%) and 5548 females (55.68%) in the panel; while specifically in the CKB and 1KGP cohort, the percentages of males were 44.29 and 50.00%, respectively. The sex distribution of the CKB individuals was highly consistent with that in the entire CKB cohort (i.e. male: 41%, female: 59%). We also provide the number of samples recruited from each sampling site in Figure 2d. Specifically, Heilongjiang, Henan and Guangxi were the top three provinces with the largest recruitments. The other provinces had relatively similar sample sizes. The sex and age distributions of samples in each sampling site are provided in Figure 2e.

We provided a comprehensive comparison in terms of sample size, averaged sequencing depth, number of variants and ancestries between the CKB reference panel and other four panels (Table 1). In detail, the TOPMed is the largest one with sample size 97 256, followed by the ChinaMAP and CKB with ∼10 000; the extended 1KGP and NyuWa included ∼3000 individuals. The sequencing depth is either medium coverage (10–30×) or high coverage (>30×). The TOPMed panel has 308.11 million variants, including 286.07 million SNPs and 22.04 million INDELs. The CKB panel had 129.74 million variants, including 113.73 million SNPs and 16.01 INDELs. The ChinaMAP, extended 1KGP, and NyuWa performed variant filtering from database to reference panel. Specifically, the ChinaMAP panel involved SNPs only (59.01 million). The extended 1KGP panel included 70.77 million variants, while SNPs counted 87.21%. The NyuWa panel had 19 million variants. By contrast, the CKB reference panel had relatively large sample sizes and detected variants compared with other panels.

**Table 1.** The information of CKB and other reference panels

Reference panel	Sample size	Sequencing depth	Variants	SNP	INDEL	Ancestries
CKB	9964	15.41×	129 743 542	113 731 044	16 012 498	Chinese
ChinaMAP	10 155	40.8×	590 10 860	59 010 860	0	Chinese
Extended 1KGP	3202	34×	707 68 225	61 715 567	9 052 658	Multiple ancestries
TOPMed	97 256	>30×	308 107 085	286 068 980	22 038 105	Multiple ancestries
NyuWa	2902	26.2×	19 256 267	-	-	Chinese

In addition, we calculated three quality indicators for SNPs: the heterozygous:homozygous (het:hom) ratio, the transition:transversion (Ti:Tv) ratio and the non-reference genotype concordance rate (NRC). The het:hom ratio is highly dependent on ancestry and the median value for Asians is ∼1.4 (42). The Ti:Tv ratio reflected the quality of SNP calling and the expected ratio would be close to 2.0 for human WGS data (27). For the CKB reference panel, we obtained a het:hom ratio of 1.31 and a Ti:Tv ratio of 1.97, indicating the high quality of genotypic data in the constructed panel. The NRC is genotype-aware recall (also known as sensitivity = TP/(TP + FN)). We used the genotype data of 50 1KGP samples with high-depth sequencing as actual status and their SNP calls in the CKB panel as the predicted data. The NRC for these 50 samples were calculated before and after genotype phasing implemented by Beagle v.5.2 (41). The average NRC increased from 0.9811 to 0.9927 and the improvement is more significant for samples with lower sequencing depth (Supplementary Figure S1a).

The PCA of individuals’ genotype data in the CKB reference panel is provided in Supplementary Figure S1b. The first PC represents a latitudinal gradient, from north to south China. As expected, individuals in the CKB reference panel were sampled from 10 different regions.

### Novel variants and variant annotation

We defined novel variants that were not assigned a unique variant accession identifier (RS number) in dbSNP (Single Nucleotide Polymorphism Database, build 154) (43). Thereby, the number of novel SNPs and INDELs are 50.16 million (44.1%) and 5.42 million (33.8%), respectively (Supplementary Figure S2a and b). Note that, a site with different mutation variety compared to that in dbSNP (e.g. in panel: REF:ALT is A:-, while in dbSNP REF:ALT is CA:C) was also considered as a novel variant, which partially explained the relatively high proportion of novel sites (44,45). As expected, most novel SNPs (99.99%) and INDELs (99.15%) were rare variants (MAF < 0.5%).

Based on the results of VEP annotation analysis, 55% were intronic variants and 26% variants located in the intergenic region. The subsequent categories were non-coding variants (15%), upstream/downstream transcript variants (12%), regulatory variants (4%), variants in mRNA untranslated regions (1%), functional variants (0.8%), transcription factor binding sites (0.3%) and splice-site variants (0.1%) (Supplementary Figure S2c). Among the functional variants, the most abundant class is missense mutation. Based on the ClinVar annotation results, there were 1604, 411, 516, 83, 12 and nine pathogenic variants for AC = 1, AC = 2, AF⇐0.1, AF⇐1, AF⇐5 and AF > 5%, respectively (Supplementary Figure S2d). Specifically, there were nine common pathogenic variants (i.e. alternate allele AF > 5%), including seven single nucleotide variation (SNV), one insertion (INS) and one deletion (DEL) (Table S2). The seven SNVs included rs7417106 (A > G, AF = 0.9468, gnomAD.EAS AF = 0.9429), rs5082 (G > A, AF = 0.9229, gnomAD.EAS AF = 0.9049), rs2280789 (A > G, AF = 0.3531, gnomAD.EAS AF = 0.3285), rs2280788 (G > C, AF = 0.1182, gnomAD.EAS AF = 0.1117), rs3754413 (C > T, AF = 0.0737, gnomAD.EAS AF = 0.0731), rs72474224 (C > T, AF = 0.0522, gnomAD.EAS AF = 0.0854) and rs77592601 (C > T, AF = 0.0510, gnomAD.EAS AF = 0.0479). In correspondence to these SNVs, the ClinVar annotated diseases included renal tubular epithelial cell apoptosis, familial hypercholesterolemia, human immunodeficiency virus type 1, rare genetic deafness, myeloproliferative neoplasm and premature rupture of membranes. The INS and DEL corresponded to hepatocellular carcinoma. The SIFT and PolyPhen algorithms provided consistent prediction of deleterious variants, that was a large fraction (96%) were very rare variants (AC⇐2) (Supplementary Figure S2e). Over 72% variants can be predicted as deleterious by both algorithms (Supplementary Figure S2f). For the low-frequency and common variants (MAF > 0.005), 23 (0.3%) of them were annotated as deleterious. In particular, seven variants were predicted as deleterious mutations by both SIFT and PolyPhen algorithms, seven variants were uniquely annotated by SIFT and nine were uniquely annotated by PolyPhen (Supplementary Table S3).

### Imputation performance evaluation

**Figure 3.**
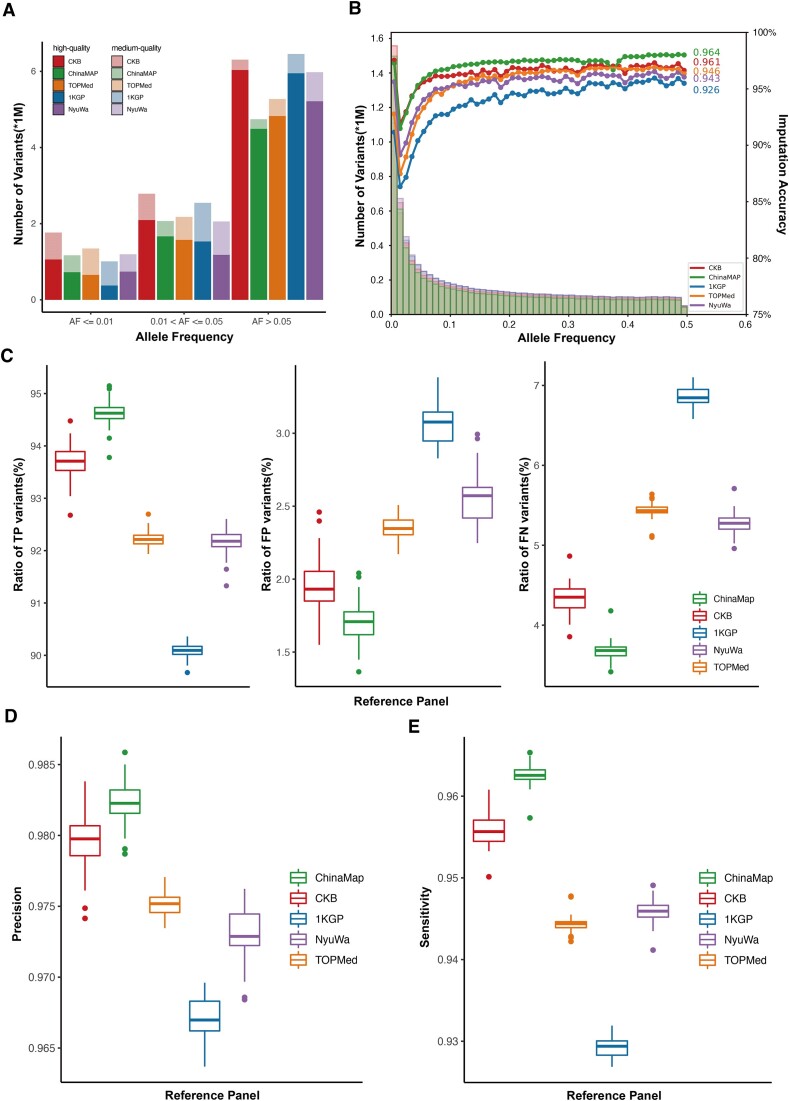
The performance for imputing 50 CKB microarray data. (**A**) The numbers of high- and medium-quality imputed variants under different AF (allele frequency) by using different reference panels. (**B**) The histogram of imputed variants and Pearson correlation coefficients for different panels. (**C**) The boxplots of the ratios of true positive (TP), false negative (FN) and false positive (FP) variants. (**D**) The imputation precision of reference panels. (**E**) The sensitivity of the reference panels.

We compared the imputation performance of the CKB reference panel with that of the extended 1KGP (6), TOPMed (46), ChinaMAP (14) and NyuWa (47) from the perspective of number of imputed variants and imputation accuracy. We used 50 CKB and 50 1KGP microarray datasets as input samples to be imputed. The corresponding high-coverage WGS data were used as ground truth datasets. In imputation of the CKB array data, the CKB reference panel provided the highest number of medium-quality imputed variants (10.86 million), followed by the extended 1KGP (10.01 million), NyuWa (9.23 million), TOPMed (8.80 million) and ChinaMAP (7.98 million) reference panels. When focusing on only high-quality imputed variants, we observed that the ChinaMAP reference panel had the greatest percentage of high-quality variants (86.23%), followed by CKB (84.63%), TOPMed (80.22%), extended 1KGP (78.50%) and NyuWa (77.32%) (Figure 3a, Table 2). We note that the reason why the ChinaMAP provides the smallest number of medium-quality variants is that it automatically filters out almost half of low-quality variants in the actually used panel.

**Table 2.** The high-quality and medium-quality imputed variants for imputing 50 microarray samples

Reference panels	Type	AF ≤ 1%	1% < AF ≤ 5%	AF > 5%	ALL
CKB	medium-quality (M)	0.71	0.69	0.27	1.67
	high-quality (M)	1.06	2.10	6.04	9.19
	high-quality rate	0.6000	0.7519	0.9570	0.8463
ChinaMAP	medium-quality (M)	0.44	0.40	0.25	1.10
	high-quality (M)	0.73	1.67	4.49	6.88
	high-quality rate	0.6205	0.8052	0.9468	0.8623
1KGP	medium-quality (M)	0.63	1.01	0.51	2.15
	high-quality (M)	0.38	1.53	5.95	7.86
	high-quality rate	0.3730	0.6021	0.9217	0.7850
TOPMed	medium-quality (M)	0.69	0.60	0.45	1.74
	high-quality (M)	0.66	1.58	4.83	7.06
	high-quality rate	0.4867	0.7234	0.9154	0.8022
NyuWa	medium-quality (M)	0.45	0.88	0.76	2.09
	high-quality (M)	0.74	1.18	5.22	7.14
	high-quality rate	0.6202	0.5741	0.8723	0.7732

Notes: (M) represents million.

We evaluated the imputation accuracy by using three measurements: Pearson correlation coefficient (*R*^2^), precision and sensitivity. The mean *R*^2^ of the compared reference panels were 0.964 (ChinaMAP), 0.961 (CKB), 0.946 (TOPMed), 0.943 (NyuWa) and 0.926 (extended 1KGP) (Figure 3b). For the ratios of true positive (TP), false positive (FP) and false negative (FN) variants, the ChinaMAP reached the highest ratio of TP variants (94.62%), subsequently followed by CKB (93.71%), then followed by TOPMed (92.21%), NyuWa (92.18%) and extended 1KGP (90.09%). Meanwhile, the ChinaMAP obtained the lowest ratios of FP (1.71%) and FN (3.68%) variants, and for the CKB panel, the two ratios were 1.93 and 4.35%, respectively. These ratios in TOPMed (FP: 2.35 and FN: 5.43%) and NyuWa (FP: 2.57% and FN: 5.28%) were slightly higher than those in the CKB panel. The extended 1KGP reference panel had the highest ratio of FP (3.08%) and FN (6.85%) variants (Figure 3c). Consequently, the ChinaMAP attained the highest precision of 98.23%, followed by CKB (97.98%), TOPMed (97.52%), NyuWa (97.29%) and extended 1KGP (96.70%). For sensitivity, the ChinaMAP and CKB panels reached 96.25 and 95.57%, respectively. Following that, the NyuWa, TOPMed and extended 1KGP obtained sensitivities of 94.59, 94.44 and 92.94%, respectively. The CKB reference panel achieved very similar *R*^2^, precision and sensitivity compared to the ChinaMAP, displaying an outstanding imputation performance (Figure 3d and e).

In the imputation of the 1KGP array data, we compared the performance of the CKB panel with that of ChinaMAP and TOPMed. We excluded the extended 1KGP panel as it had overlap samples with the array data, and also excluded NyuWa panel as the web server is unstable and not accessible for submitting jobs currently. The CKB reference panel provided the highest number of medium-quality imputed variants (9.75 million), followed by TOPMed (7.83 million) and ChinaMAP (6.98 million) reference panels. When focusing on only high-quality imputed variants, we observed that the ChinaMAP reference panel had the greatest percentage of high-quality imputed variants (87.92%), followed by TOPMed (84.46%) and CKB (84.11%) (Supplementary Figure S3a). For the Pearson correlation coefficient *R*^2^, both the CKB and ChinaMAP panels achieved 0.979, while the TOPMed had a lower *R*^2^ of 0.965 (Supplementary Figure S3b).

### Imputation of 100 706 microarray data

**Figure 4.**
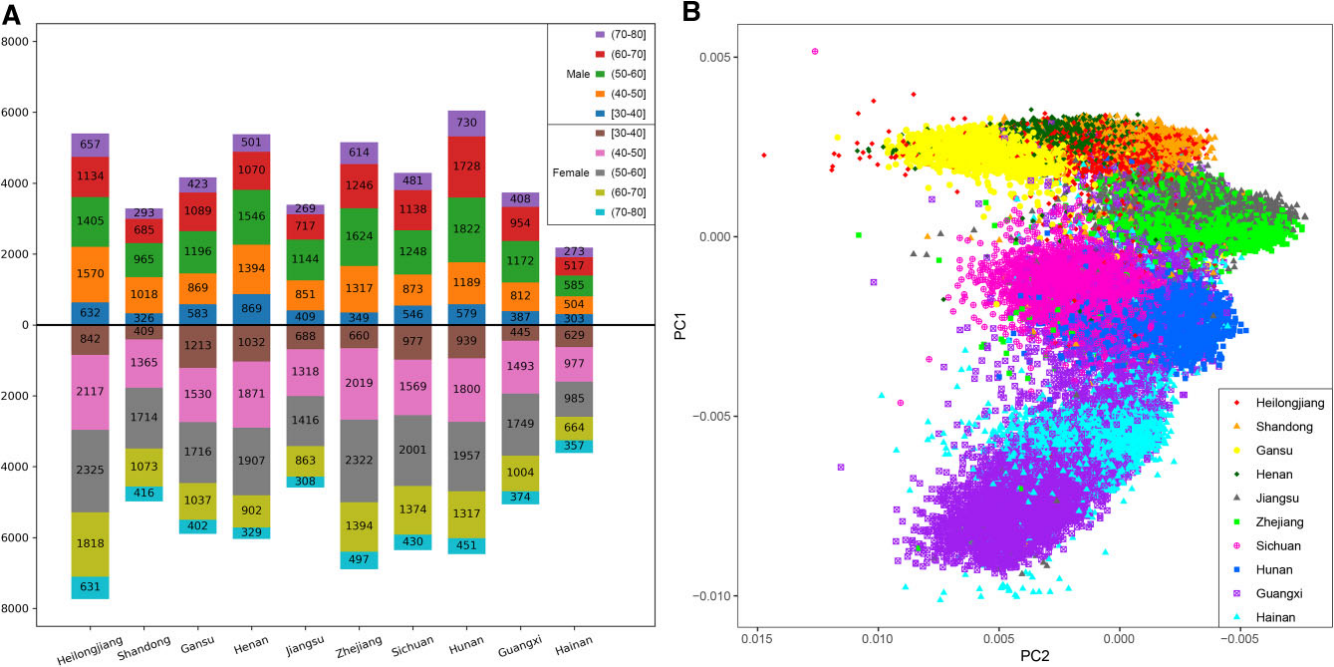
The sample information and principal component analysis of the microarray data. (**A**) The sex and age distribution of samples in each sampling site. The age distributions of males (females) were on the top (bottom) of the *x*-axis. The total number of samples from all sampling sites was 100 640, as 66 samples with missing sampling site information. (**B**) The principal component analysis of 100 706 samples with microarray data before genotype imputation. The PC1 represents a latitudinal gradient, from north to south China. Each color represents a province of sampling site.

For the 100 706 samples with microarray data, we provided their sex and age distribution in each sampling site (Figure 4a). Specifically, the provinces of Heilongjiang (*N* = 13 131), Hunan (*N* = 12 512), Zhejiang (*N* = 12 042), Henan (*N* = 11 421), Sichuan (*N* = 10 637) and Gansu (*N* = 10 058) had recruitments >10 000. The province of Hainan had smallest recruitment of 5794. The PCA of microarray data before imputation was provided in Figure 4b. The PC1 represents the latitudinal gradient. The imputation-completed whole genome data contained 42.61 million medium-quality variants and 17.45 million high-quality variants. To assess the imputation performance of the 100 706 CKB microarray data, we calculated the Pearson correlation coefficients (*R*^2^) of 50 CKB samples with imputed genotype and high-depth WGS data. Note that we did not have WGS data for the 100 706 samples, thus we could not use that as the true set. As an alternative, we used a subset of 50 individuals with WGS data as samples being evaluated. Consequently, the averaged *R*^2^ was 0.972. Remember that when we simulated only these 50 microarray samples, the averaged *R*^2^ was 0.961 (Supplementary Figure S4). This *R*^2^ difference may be due to the randomity of the imputation algorithm in Beagle v.5.2, hidden Markov model (28). The high imputation accuracy of 0.972 demonstrated that the proposed CKB reference panel is quite capable of imputing extensive data.

### GWAS analysis of simulated data

With imputed phenotype data under the null hypothesis that there were no associated SNPs, the GWAS analysis did not identify any significant signals and the *P*-values were uniformly distributed (Supplementary Figure S5a and b) as expected. When the phenotype was generated by involving the effects of SNPs, the GWAS study successfully discovered causal SNPs and those in high linkage disequilibrium (LD) (Supplementary Figure S5c). Specifically, in addition to the five randomly selected causal SNPs (rs3003378, rs6764623, rs10905649, rs13254191 and rs10915307), high-LD SNPs (e.g. rs12564681, rs11923809, rs7092291, rs545854, rs12123277) were also identified. The results of GWAS analysis with simulated data under both null and alternative hypotheses demonstrated the high-quality of genotype data.

### GWAS analysis of real phenotype data

**Figure 5.**
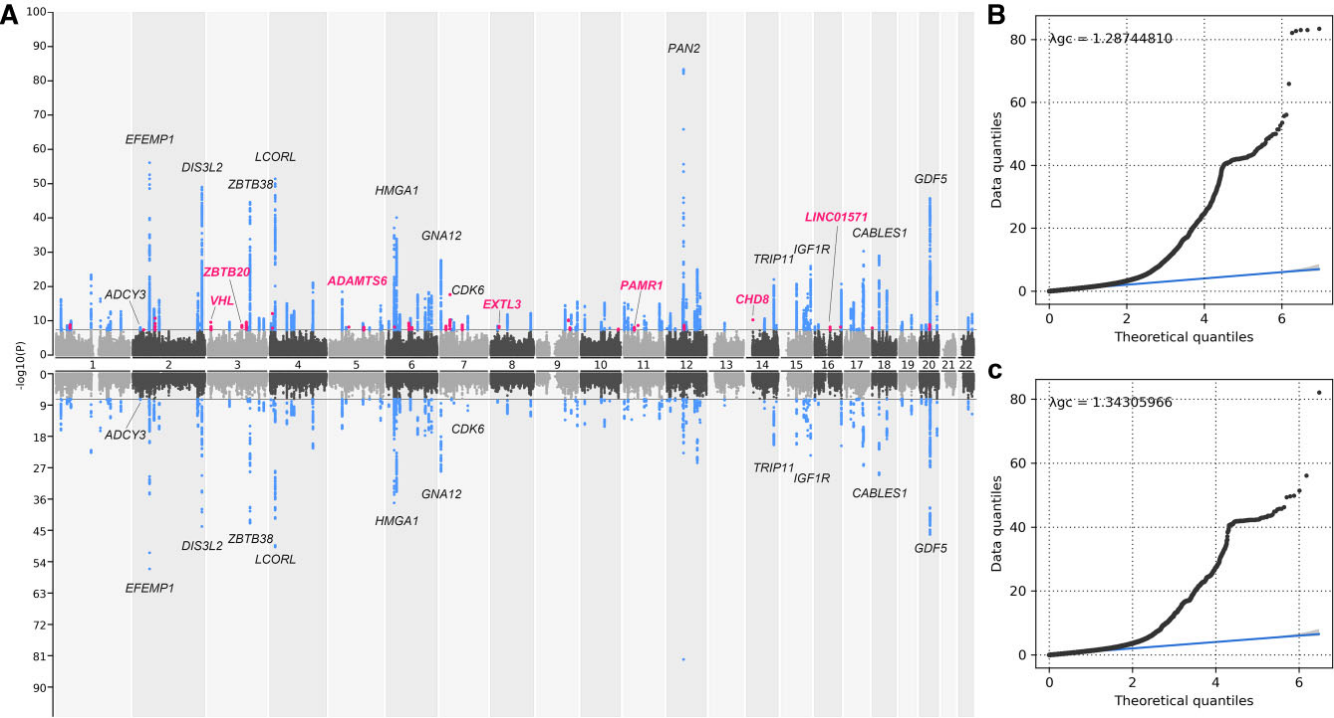
The GWAS results of height before and after genotype imputation. (**A**) The mirrored Manhattan plots of GWAS results based on the microarray data after (top) and before (bottom) genotype imputation. Genes in black are a list of shared genes identified before and after imputation. Genes in purple are a list of representative genes identified only after imputation. (**B**) The QQ-plot of GWAS results after genotype imputation. (**C**) The QQ-plot of GWAS results before genotype imputation. The genomic inflation factors (*λ*_gc_) were 1.287 and 1.343 after and before imputation, respectively.

After filtering in SNPs with MAF > 0.01, HWE *P*-value >1E-06, and genotype missing rate <0.01, the numbers of SNPs in GWAS analysis before and after imputation were 3 038 178 and 9 205 896, respectively. The increase in number of SNPs was substantial. At the significance threshold of 5E-08, the number of significant SNPs increased from 7971 to 16 508 after imputation (Figure 5, Supplementary Table S4). The numbers of identified significant loci for original and after-imputed data were 119 and 147, respectively. The shared 119 loci included the well-known height-associated genes *GDF5* (cartilage-derived morphogenetic protein 1) (48), *IGF1R* (insulin-like growth factor 1 receptor) (49), and *ADCY3* (ATP pyrophosphate-Lyase 3) (50). Among the additional 28 loci, 26 (92.9%) were previously reported to be associated with height, for example *CHD8* (chromodomain helicase DNA binding protein 8) functioned in transcriptional regulation and promotion of cell proliferation (51), *ZBTB20* (zinc finger protein 288) played a role in glucose homeostasis and postnatal growth (51), and *PAMR1* (regeratioin-associated muscle protease homolog) might played a role in regeneration of skeletal muscle (51). The GWAS results with real phenotype indicated the high quality and credibility of the imputed data.

## Discussion

A population-specific haplotype reference panel is a collection of ancestral chromosome sequences that represents the genetic diversity of the population. A high-precision reference panel is the basis for population genetic analysis and precision medicine. China has the largest population in the world and possesses vast amounts of genetic resources, but lacks a high-quality reference panel, which has hindered the development of genetic studies and their application in human diseases based on the Chinese population. Fortunately, in the last 2 years, a few reference panels have been constructed for accurate genotype imputation in the Chinese population, including the ChinaMAP and NyuWa.

In this work, we developed a high-resolution haplotype-resolved reference panel of 10 000 sequenced individuals from the CKB cohort and the 1KGP database. Even with medium sequencing depth (15.41×), the proposed CKB panel can compete with the ChinaMAP (40.80×) and outperform the extended 1KGP, TOPMed and NyuWa in imputation accuracy measured by Pearson correlation coefficient, precision and sensitivity. From the perspective of the number of well-imputed variants, the CKB provided the largest number of medium-quality variants with an information score between 0.4 and 0.8; for high-quality variants with an information score >0.8, the CKB panel obtained the second largest amount among all considered panels. What is more valuable is that we completed the genotype imputation for 100 706 CKB microarray data based on the constructed panel. The imputation accuracy reached as high as 0.972 and GWAS analysis based on the simulated data and the real phenotype height demonstrated the reliability of the extensive imputed data. This imputed dataset is the largest whole genome data for Chinese population to date and will certainly play a fundamental role in personalized medicine and drug development.

However, it must be acknowledged that our study has some limitations. First, the sequencing depth is medium (∼15×). Based on our evaluation, compared to high coverage data (>30×), medium sequencing data have comparable base quality measured by Q20, Q30 and GC content. However, the genomic coverage at different sequencing depth has differences, especially for higher coverage. In detail, for 1×, 4× and 10×, the coverage differences are about 0.2, 1.0 and 18%, respectively, which might have influence on rare and novel variants detection. We note that the comparison results were obtained from two particular datasets and could not represent a general tendency. Second, 9914 out of 9964 (99.50%) subjects in the CKB reference panel were stroke cases, even though the results of variants detection and association analysis were promising, the explicit influence of potential disease haplotype is hard to tell and needs further investigation.

The ultimate goal of imputing genotype data is to increase statistical power of genetic association studies for identifying trait-associated SNPs and to reveal the etiology of complex diseases. As the hitherto largest cohort of Chinese population, CKB collected abundant clinical data, including demographic, anthropometric, biochemical, radiographic traits, metabolomic tests and diseases coded by ICD10 (international classification of diseases, v.10). There are >1500 diseases, mostly chronic, such as heart attack, stroke, diabetes, cancers and so on. As a significant future work, we aim to perform GWAS analysis for the vast wealth of phenotypes and over 100 000 imputed WGS genotype data. In recent years, as a precision medicine tool, the polygenic risk score, also known as the polygenic score, has been widely used to predict an individual's genetic risk of disease. The predictive accuracy of the polygenic risk score largely relies on the sample sizes in discovery samples. To the best of our knowledge, with the after-imputed genomic data, it should be the largest population genetic study of the Chinese population and is also comparable to numerous international genomics research projects, for example, the UK Biobank study (https://www.ukbiobank.ac.uk/), the All of Us research program (https://allofus.nih.gov/) and the biobank Japan project (https://biobankjp.org/en/).

Most of the reference panels are now packaged into online imputation servers, such as the Michigan imputation server (40), TOPMed imputation server (40), ChinaMAP imputation server, NyuWa server and our developed CKB imputation server. These imputation servers all provide free genotype imputation service by uploading to-be-imputed files and selecting reference panel, population and imputation software. All the imputation results can be downloaded directly by clicking on filenames. Even though the online server provides a convenient way to impute genotype data, it typically cannot handle large-sized files, which causes difficulties in imputing large-sample data. When imputing large-scale datasets, the individual-level reference panels are needed for offline imputation. Since the completion of the first human genome project in 2003 (https://www.genome.gov/human-genome-project), the only database that is fully publicly available is the 1000 Genomes Database. Sharing genomic data is critical for research efficiency, translating research results into clinical applications and ultimately improving public health. Hence, we appeal for the sharing of genomic and health-related data with controlled management.

## Supplementary Material

gkad779_Supplemental_Files

## Data Availability

The CKB reference panel and the after-imputed >100 000 CKB microarray data have been deposited into CNGB Sequence Archive (CNSA) of China National GeneBank DataBase (CNGBdb) with accession number CNP0003405. All genotype data are shared with controlled management.
