# Supplementary material for: A high-resolution haplotype-resolved Reference panel constructed from the China Kadoorie Biobank Study
Source: Nucleic Acids Res. 2023 Oct 23;51(21):11770–82. doi: 10.1093/nar/gkad779 (PMC10681741; doi:10.1093/nar/gkad779)
Supplement: gkad779_Supplemental_Files [file gkad779_supplemental_files.zip › 04-Supplementary Figures-.pdf]

A High-resolution Haplotype-resolved Reference Panel Constructed from the China Kadoorie Biobank Study

Supplementary figures

Figure S1. The concordance information and principal component analysis of the CKB reference panel.

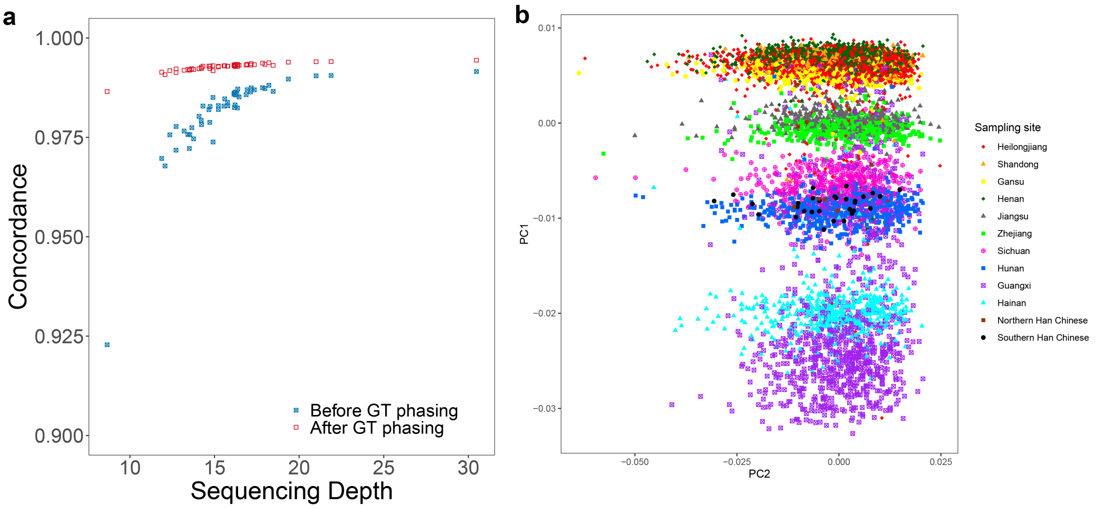

Notes: (**a**) The non-reference concordance rate before and after genotype phasing versus sequencing depth. (**b**) The principal component analysis of the CKB reference panel. The PC1 represents a latitudinal gradient, from north to south China. Each color represents a province of sampling site.

Figure S2. The sequence information about the CKB reference panel.

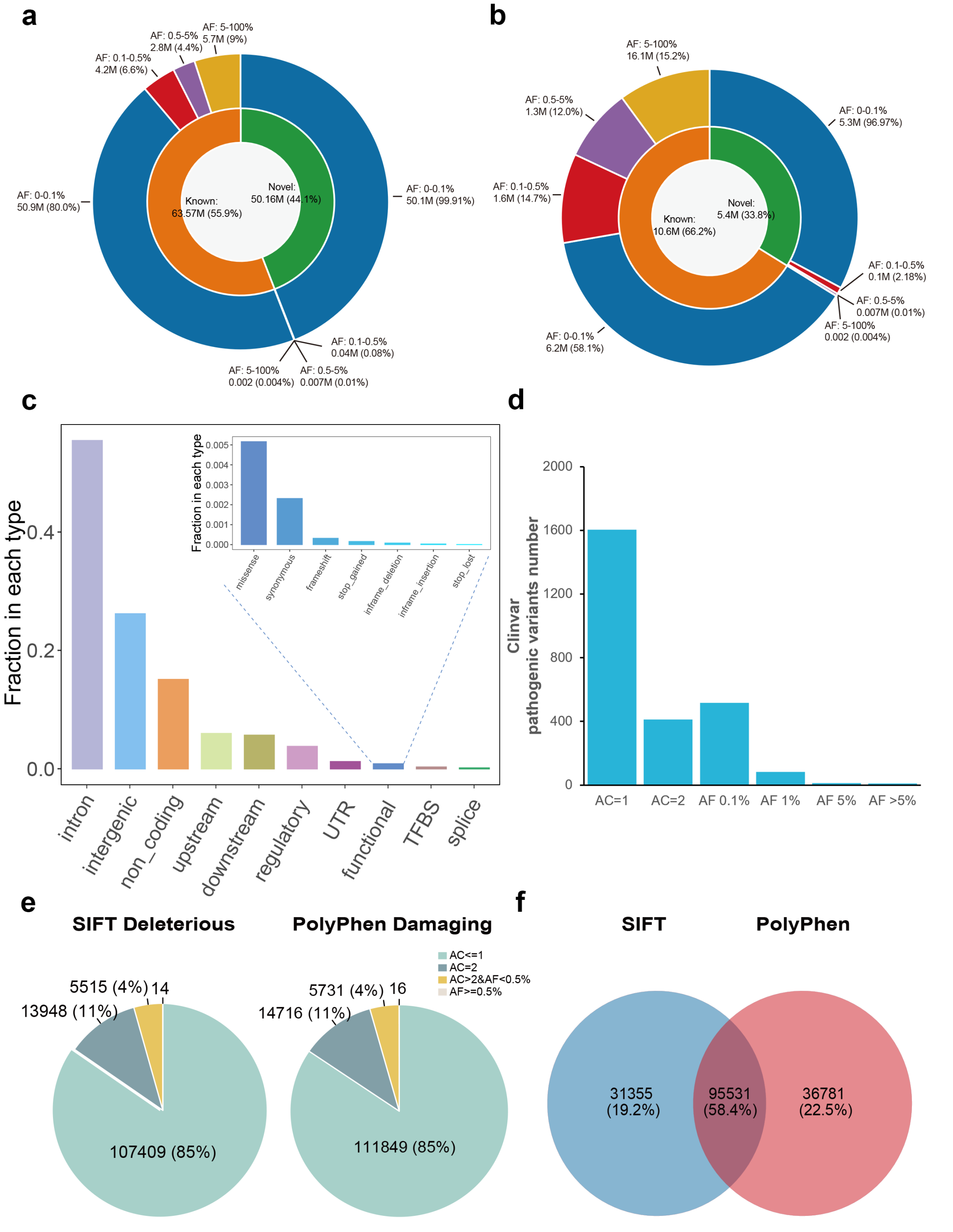

Notes: (**a**) The inner circle showed the number and proportion of the novel and known SNPs included in the CKB reference panel. The outer circle showed the number and proportion of SNPs with different AFs. (**b**) The inner circle showed the number and proportion of the novel and known INDELs included in the CKB reference panel. The outer circle showed the number and proportion of INDELs with different AFs. (**c**)

Annotations of all variants. The major bar chart showed all the 10 categories. The minor bar chart displayed the functional variants with more detailed roles. (**d**) The number of pathogenic variants according to different allele count (AC) and allele frequency (AF) annotated by ClinVar. (**e**) Distribution of novel deleterious variants annotated by SIFT (left) and damaging variants annotated by PolyPhen (right) for different allele counts or frequencies. (**f**) The Venn diagram of numbers of novel deleterious variants annotated by SIFT and PolyPhen.

Figure S3. The performance for imputing 1KGP microarray data.

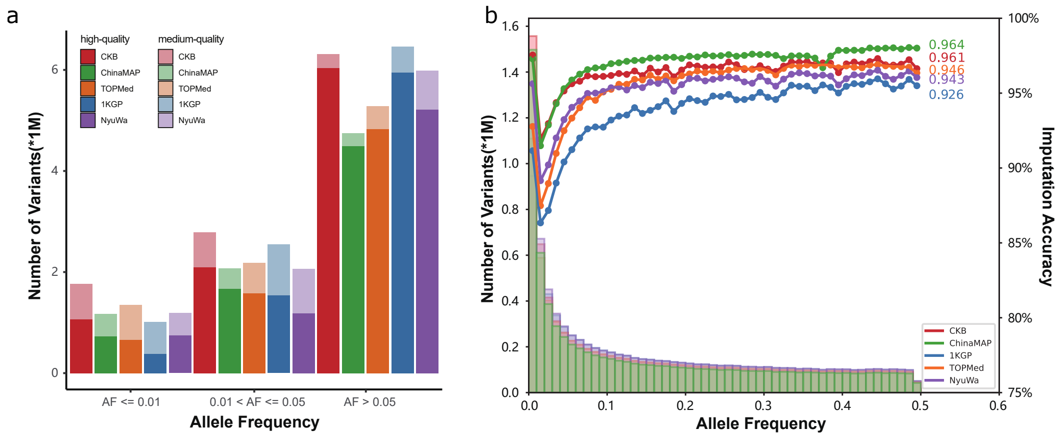

Notes: (**a**) The numbers of high- and medium-quality imputed variants under different AF (allele frequency) by using different reference panels. (**b**) The histogram of imputed variants and Pearson correlation coefficients for different panels.

Figure S4. The imputation performance for imputing 100,706 microarray samples.

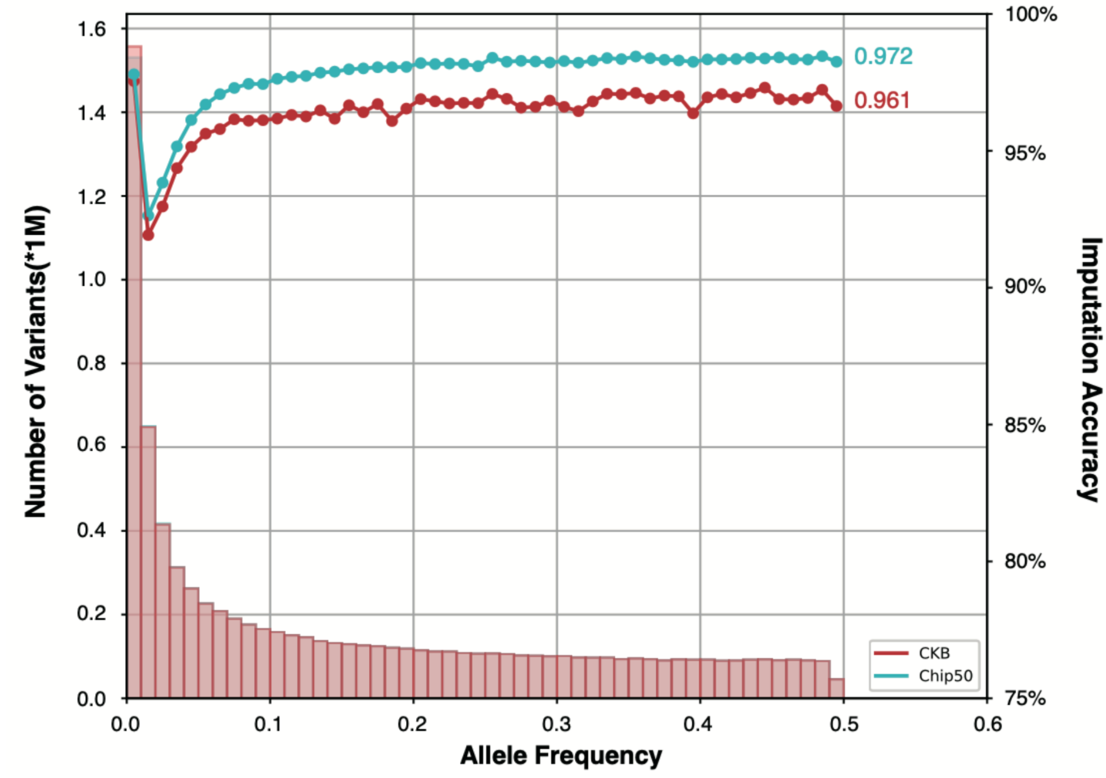

Notes: The Pearson correlation coefficient was calculated between the true high coverage WGS data and 50 microarray data either extracted from the entire 100,706 after-imputed dataset (green) or imputed alone (red).

Figure S5. The GWAS results of simulated phenotypic data.

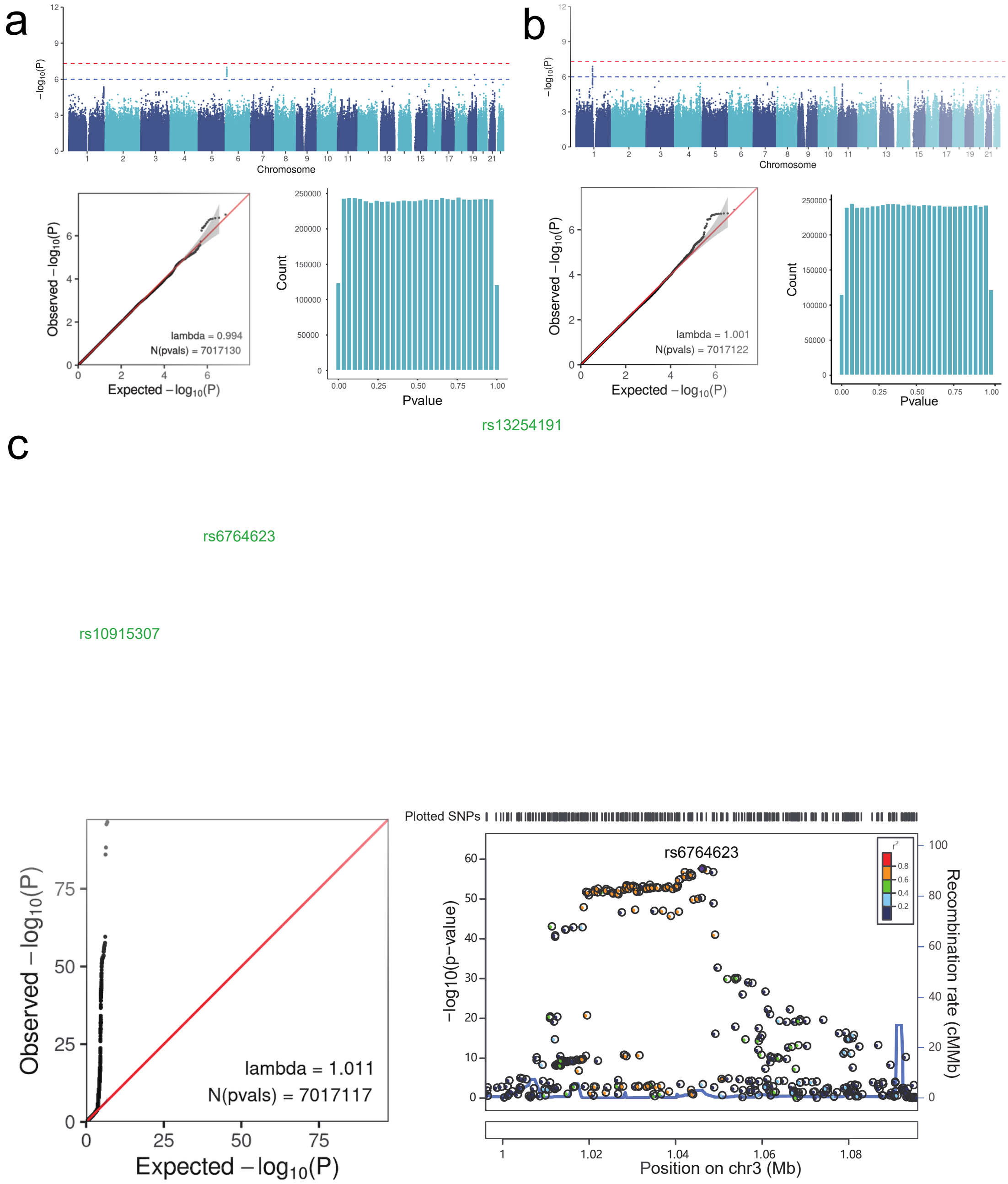

Notes: (**a**) The GWAS results with simulated binary phenotypic data under the null hypothesis. (**b**) The GWAS results with simulated quantitative phenotypic data under the null hypothesis. (**c**) The GWAS results of simulated phenotypic data under the alternative hypothesis.
